# Significance of molecular and zymography profiling for accurate detection of infected sheep with *Anaplasma marginale* in South Sinai, Egypt

**DOI:** 10.1038/s41598-025-19665-5

**Published:** 2025-10-07

**Authors:** Manar A Sayed, Abdel-Rahman B. Abdel-Ghaffar, Sayed M. M. Abd El Baky, Huda O. AbuBakr, Said S. Moselhy, Rehab Abdel-Hameed

**Affiliations:** 1 Animal Health Department, Animal and Poultry Production Unit, Desert Research Center, Cairo, Egypt; 2 Biochemistry Department, Faculty of Science, Ain Shams University, Cairo, Egypt; 3 Biochemistry& Molecular Biology Department, Faculty of Veterinary Medicine, Cairo University, Giza, Egypt

**Keywords:** Anaplasmosis, Sheep, Oxidative stress, DNA methyltransferase 3A, MSP-5, Southern sinai, Biochemistry, Ecology, Molecular biology

## Abstract

*Anaplasma marginale* is a significant tick-borne pathogen affecting ruminants; yet its prevalence and pathological impact in sheep remain underreported. This study aimed to investigate the prevalence of *A. marginale* in sheep from Southern Sinai, Egypt, along with its molecular characterization and the associated oxidative stress and immune responses. Microscopic examinations and PCR targeting the msp4 and msp5 genes were employed for detection, with sequencing and phylogenetic analysis performed on the msp5 gene. Oxidative stress markers, including malondialdehyde (MDA), superoxide dismutase (SOD), and glutathione (GSH), were quantified using commercial kits. Inflammatory response was assessed via gelatin zymography for matrix metalloproteinase (MMP-2 and MMP-9). Expression levels of candidate genes, DNA methyltransferase 3 A (DNMT3a), IL-1β, and heat shock proteins (HSPs) were measured by qPCR. A high prevalence of *A. marginale* infection was observed, with 76% positivity by microscopy and 79.85% by PCR. Sequencing of the msp5 gene revealed 95.2–100% identity with global strains, clustering closely with the Florida (USA) strain in the phylogenetic tree. Infected sheep exhibited significantly elevated MDA levels (*p* < 0.05) and decreased SOD and GSH levels, indicating oxidative stress. MMP-2 and MMP-9 activities were also significantly increased, suggesting inflammatory involvement. Moreover, qPCR revealed significant upregulation of DNMT3a, IL-1β, and HSPs, highlighting transcriptional changes associated with infection. These findings provide valuable insights into molecular epidemiology and host response to *A. marginale* in sheep. The combination of msp5 sequencing, oxidative stress biomarkers, zymographic profiles, and gene expression analysis of DNMT3a, IL-1β, and HSPs suggests a potential panel of biomarkers for the diagnosis and monitoring of ovine anaplasmosis.

## Introduction

The Anaplasmataceae family comprises obligate intracellular bacteria, including the genus *Anaplasma*, which is part of the order *Rickettsiales*. Although *Anaplasma* is phylogenetically related to *Rickettsia*, it is taxonomically distinct and belongs to a different family (*Rickettsiaceae* vs. *Anaplasmataceae*). These pathogens are primarily transmitted by arthropods and are responsible for significant endemic and emerging infectious diseases in both humans and animals, posing substantial economic and public health concerns^1^.

Anaplasmosis, in particular, leads to considerable economic impact due to its high morbidity and mortality rates, reduced animal productivity, and the financial burden associated with treatment and disease control. The pathogen exhibits marked variation in biology, morphology, protein sequences, and antigenic characteristics according to geographical distribution^2–4^. Although the disease primarily affects cattle, it can also infect buffalo, sheep, goats, and wild ruminants^5,6^.

More than 20 species of ixodid ticks, including *Dermacentor spp*. and *Rhipicephalus spp*., have been identified as vectors of *Anaplasma spp*^7^. Among the known *Anaplasma species*, *A. marginale* is the most virulent, characterized by severe hemolytic anemia. This tick-borne, obligate intraerythrocytic pathogen is the primary causative agent of bovine anaplasmosis and has also been implicated in infections in small ruminants^8^. Molecular detection of *A. marginale* has been reported in sheep, camels, and *Hyalomma dromedarii* ticks infesting camels^9,10^.

Previous biochemical studies revealed that circulating cytokines stimulate the release of acute-phase proteins (APPs) from the liver, playing a crucial role in the innate immune response^11^. Blood levels of APPs often correlate with the severity of inflammation or infection and therefore could serve as reliable biochemical indicators of the inflammatory response^12,13^.

The heat shock and other stress responses are conserved cellular mechanisms activated under elevated temperatures, toxicity, and pathogenic infection. Heat-shock proteins (HSPs) such as HSP70, and other stress response proteins play protective roles by preserving cellular integrity and restoring normal physiological functions. At the molecular level, the heat-shock response is a transient reprogramming of cellular activities mediated by the synthesis of HSPs. Extracellular and membrane-bound HSPs also function in antigen binding and immune system activation^14^.

Oxidative stress and inflammation play a pivotal role in the pathogenesis of the disease. Studies have shown that reactive oxygen and nitrogen species, including nitric oxide and hypochlorous acid, along with key inflammatory mediators such as IL-1β and TNF-α, can modulate the activity of matrix metalloproteinases (MMPs). MMPs are a prominent family of zinc-dependent extracellular proteases that contribute to tissue remodeling during infection^15,16^.

This study was conducted to improve the molecular diagnosis of *A. marginale* infection in sheep, enabling timely intervention to reduce economic losses in livestock. The specific objectives were: first, to generate current geo-epidemiological data and public health strategies regarding anaplasmosis prevalence in specific regions of Egypt; second, to identify *Anaplasma* species at the molecular level in naturally infected small ruminants. This identification was performed by targeting the msp4 and msp5 genes, which belong to a group of six major surface protein genes (msp1a, msp1b, msp2, msp3, msp4, and msp5) commonly used to characterize Anaplasma marginale genetic diversity^17^. These genes provide an enhanced degree of specificity for *A. marginale* detection and differentiation, making them valuable molecular markers for epidemiological studies^9,17^.

The research also aimed to assess oxidative stress biomarkers in the blood of host animals, characterize the immune response to *Anaplasma* infection, and ultimately, to elucidate how Anaplasmosis triggers transcriptional changes that alter host cell functions.

Although molecular tools such as PCR and sequencing are widely used in the diagnosis of anaplasmosis, their application in small ruminants, particularly sheep, remains limited in the Egyptian context. Given the regional differences in vector ecology and pathogen diversity, validating these diagnostic tools locally is essential for accurate disease surveillance and control. This study contributes novel epidemiological and molecular data on A. marginale in sheep from underrepresented areas such as Ras Sudr and Al-Tur in Sinai, enhancing the national database on ovine anaplasmosis.

## Materials and methods

### Animals and design of experiments

**Fig. 1:**
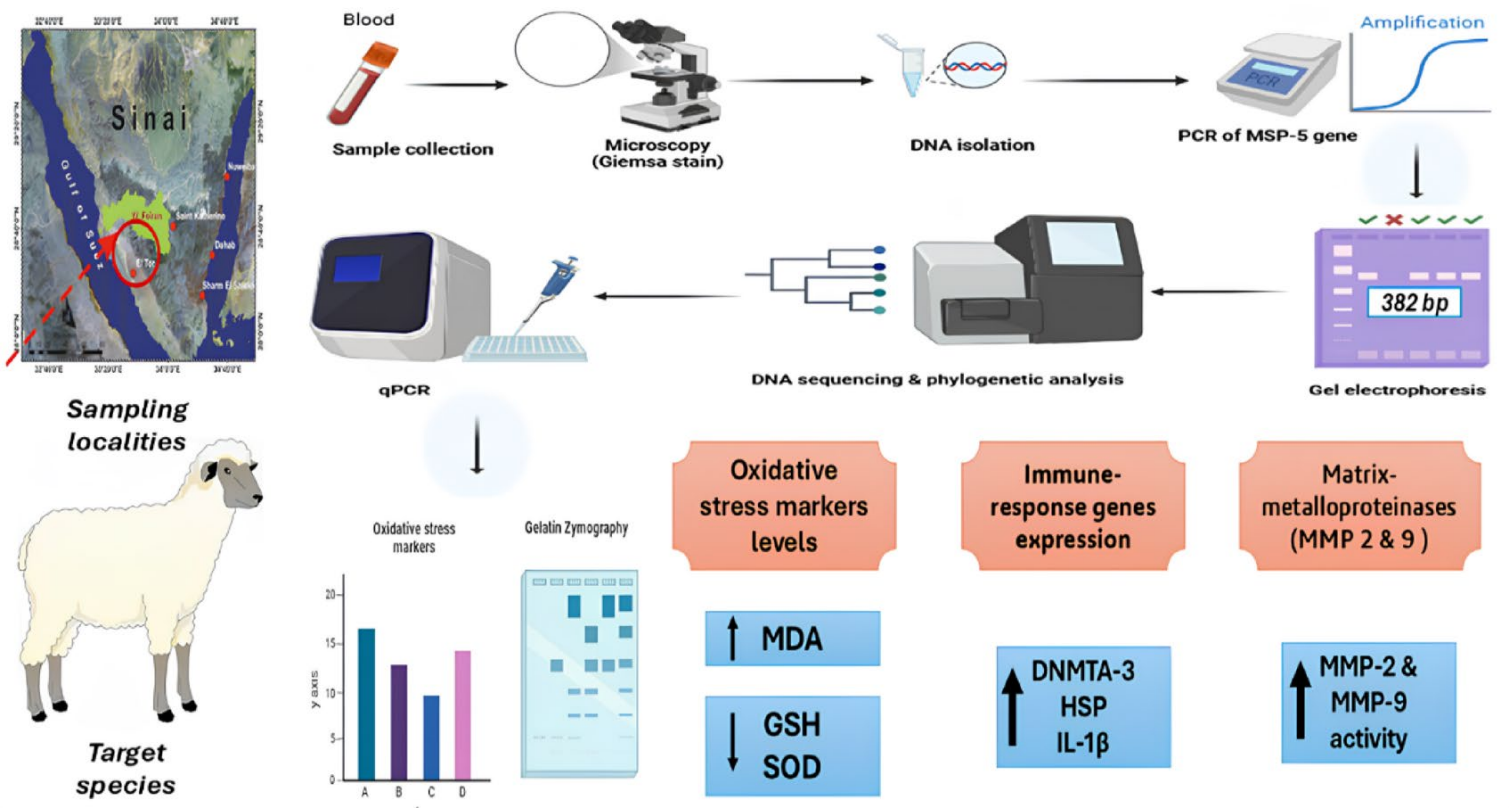
Workflow of the experimental design. Blood samples were collected from sheep in the Sinai region and examined by microscopy (Giemsa staining). DNA was isolated and the msp5 gene was amplified by PCR and confirmed by gel electrophoresis (382 bp). Positive samples were sequenced, and phylogenetic analysis was performed using reference strains from GenBank. Quantitative PCR (qPCR) was used to assess gene expression, while biochemical assays determined oxidative stress markers (MDA, GSH, SOD). Immune-response genes (DNMTA-3, HSP, IL-1β) and matrix metalloproteinases (MMP-2, MMP-9) activities were further analyzed.

The geographical scope of the current study was two sampling localities in South Sinai Governorate: Tur Sinai City and Ras Sudr research station, following Desert Research Center (DRC, Egypt. The study was conducted on 139 female Barki sheep randomly selected (from November 2020 to April 2021), with no treatment against Anaplasmosis. Ten clinically healthy animals were randomly selected and served as controls for comparison. All animal procedures were approved by the Institutional Animal Care and Use Committee (Vet. CU. IACUC) under approval number Vet CU 13102024947. The study is reported in accordance with the ARRIVE guidelines. All methods were performed in accordance with the relevant guidelines and regulations. An overview of experimental workflow is presented in Fig. 1.

### Sampling and microscopy

Blood samples were collected from the jugular vein and divided into three parts: (a) a plain tube (to separate serum for the Gelatin Zymography analysis), (b) a tube with heparin (for oxidant/antioxidant biomarkers analysis) and (c) a tube with EDTA (for preparing blood smears for staining techniques as described by^18,19^, and the remaining were stored at -80 °C for DNA and RNA extraction for molecular studies). A sample of 10 µL EDTA blood was then used to prepare thin smear slides, stained according to Giemsa’s method (May–Gr€unwald–Giemsa [MGG]), and examined under low and high powers to identify inclusion bodies consistent with anaplasmosis based on morphology and intra-erythrocytic localization^20,21^.

### Genomic DNA extraction

Genomic DNA was extracted from whole blood samples using the Biospin Whole Blood Genomic DNA Extraction Kit Cat no. BSC06N1 (Bioer Technology, China) according to the manufacturer’s instructions. Briefly, 10 µL of proteinase K solution was pipetted into a 1.5 mL microcentrifuge tube, followed by the addition of 200 µL of whole blood. Subsequently, 200 µL of Lysis B buffer was added and mixed thoroughly for 5–10 s, then incubated at 56 °C for 10 min. After incubation, 200 µL of ethanol was added and mixed gently. The mixture was transferred to a spin column and centrifuged at 6000–8000 × g for 1 min, and the flow-through was discarded. The column was then washed with 500 µL of WB1 buffer and centrifuged at 10,000 × g for 30–60 s, followed by discarding the flow-through. Next, 700 µL of wash buffer was added and centrifuged again at 10,000 × g for 30–60 s. The column was placed back into the collection tube and centrifuged at 13,000 × g for 2 min to remove residual wash buffer. Finally, the column was transferred to a clean 1.5 mL microcentrifuge tube, 100 µL of elution buffer was added, incubated at room temperature for 1 min, and then centrifuged at 13,000 × g for 1 min. The eluted DNA was stored at − 20 °C until further analysis.

### Conventional PCR amplification

The extracted DNA was examined by PCR for the detection of the *A. marginale* major surface protein 5 (*msp5*) gene, as previously described^21,22^. Genomic DNA extracted from infected animals was used as positive control, and sterile water served as the negative control. The PCR was performed using an automatic thermocycler (Bio-Rad) in a total reaction volume of 50 µL, containing 10 µl of High-yield PCR master mix, 2 µl of Forward primer, 2 µl of Reverse primer, 8 µl of DNA template and 27 µl of Nuclease free water.

The reaction mixtures were gently mixed, briefly centrifuged, and placed in a thermal cycler under the following cycling conditions. For *msp5* gene were: 4 min of initial denaturation at 94 °C, followed by 35 cycles of 94 °C for 1 min, 55 °C for 1 min, and 72 °C for 1 min, with a final extension at 72 °C for 10 min. For *msp4* amplification, the initial denaturation step was 30 s at 94 °C, followed by 35 cycles of 30 s at 94 °C, 30 s at 60 °C, and 1 min at 68 °C. PCR products were visualized by UV transillumination after electrophoresis in a 1% agarose gel stained with ethidium bromide. Primer sequences used for amplification are shown in Table 1.

Gel images were cropped to focus on the target bands for clarity in figure presentation. No lanes were removed or rearranged, and no selective modifications were applied. Unfortunately, the original uncropped gel images could not be retrieved due to data archiving limitations at the time of capture.

**Table 1:** Oligonucleotide primers used in PCR amplification.

Species	Target gene	Primer sequence (5´–3´)	Fragment length (bp)	References
*Anaplasma* *Marginale*	MSP5	F 5´-ACAGGCGAAGAAGCAGACAT-3´	382 bp	^22^
		R 5´-ATAAATGGGAACACGGTGGA-3´		
	MSP4	F 5´-CCCATGAGTCACGAAGTGG-3´	753 bp	^23^
		R 5´-GCTGAACAGGAATCTTGCTCC-3´		

### DNA sequencing and phylogenetic analysis

PCR products were purified using the QIAquick PCR Product Purification Kit (Qiagen, Valencia, CA, USA) according to the manufacturer’s instructions. Purified amplicons were sequenced in both forward and reverse directions on an Applied Biosystems 3130 automated DNA sequencer (ABI 3130, USA) using the BigDye Terminator v3.1 Cycle Sequencing Kit (Perkin-Elmer, Foster City, CA, USA; catalog number 4336817). The resulting sequences were analyzed using the BLAST^®^ tool (Basic Local Alignment Search Tool) to determine identity with GenBank reference sequences^24,25^. Additional phylogenetic analyses were conducted with maximum likelihood, neighbor-joining, and maximum parsimony algorithms implemented in MEGA6 software^26^.

### Determination of oxidative stress biomarkers

#### Malondialdehyde (MDA) concentration assay (µmol/mL)

Malondialdehyde (MDA), a marker of lipid peroxidation, was quantified using a thiobarbituric acid (TBA) reactive substances assay, as described by Lykkesfeldt^27^. In this method, TBA reacts with MDA in an acidic medium at 95 °C for 30 min to produce a pink chromogen measurable at 534 nm. Briefly, the reaction mixture was incubated, and the absorbance of each sample (A _sample_) was measured at 534 nm against a reagent blank using a UNICO UV-2100 spectrophotometer. The absorbance of the standard (A _standard_) was measured against distilled water. The concentration of MDA in serum was calculated using the following formula:

$$\:\text{M}\text{D}\text{A}\:({\upmu\:}\text{m}\text{o}\text{l}/\text{m}\text{L})=\frac{\text{A}\:\text{s}\text{a}\text{m}\text{p}\text{l}\text{e}}{\text{A}\:\text{s}\text{t}\text{a}\text{n}\text{d}\text{a}\text{r}\text{d}\:}\times\:10$$

#### Superoxide dismutase (SOD) activity assay (U/mL)

SOD activity was assessed using the method of Marklund and Marklund^28^, which is based on the enzyme’s ability to inhibit the phenazine methosulfate (PMS)-mediated reduction of nitroblue tetrazolium (NBT). Absorbance was measured at 560 nm over a period of 5 min at 25 °C using the UNICO UV-2100 spectrophotometer. The percentage inhibition of NBT reduction by SOD was calculated as follows:

$$\:\mathbf{\%}\text{I}\text{n}\text{h}\text{i}\text{b}\text{i}\text{t}\text{i}\text{o}\text{n}=\frac{{\Delta\:}\text{A}\:\text{c}\text{o}\text{n}\text{t}\text{r}\text{o}\text{l}-{\Delta\:}\text{A}\:\text{s}\text{a}\text{m}\text{p}\text{l}\text{e}}{{\Delta\:}\text{A}\:\text{c}\text{o}\text{n}\text{t}\text{r}\text{o}\text{l}}\times\:100$$

Where:

- $\:{\Delta\:}\text{A}\:\text{c}\text{o}\text{n}\text{t}\text{r}\text{o}\text{l}\:$is the change in absorbance at 560 nm in the absence of the sample.
- $\:{\Delta\:}\text{A}\:\text{s}\text{a}\text{m}\text{p}\text{l}\text{e}$ is the change in absorbance at 560 nm in the presence of the sample.

The SOD activity was calculated using:

$$\:\text{S}\text{O}\text{D}\:\text{a}\text{c}\text{t}\text{i}\text{v}\text{i}\text{t}\text{y}\:\:\text{U}/\text{m}\text{L}=\text{\%}\text{I}\text{n}\text{h}\text{i}\text{b}\text{i}\text{t}\text{i}\text{o}\text{n}\times\:\:3.75$$

#### Reduced glutathione (GSH) concentration assay (mmol/L)

GSH concentration was determined according to the method described by Turrens and Wu^29,30^. The assay is based on the reduction of 5,5’-dithiobis-(2-nitrobenzoic acid) (DTNB) by GSH to yield a yellow chromophore measurable at 405 nm. Following a (5–10) minute reaction period, the absorbance of each sample (Asample) was read against a blank at 405 nm using the UNICO UV-2100 spectrophotometer. The GSH concentration was calculated using the following equation:

$$\:\text{G}\text{S}\text{H}\:(\text{m}\text{m}\text{o}\text{l}/\text{L})=\text{A}\text{s}\text{a}\text{m}\text{p}\text{l}\text{e}\times\:2.22$$

### Assessment of matrix metalloproteinases (MMP-2 & MMP-9) activity by gelatin zymography

Gelatin zymography was performed to assess the enzymatic activity of matrix metalloproteinases MMP-2 and MMP-9, following the protocol described by Hawkes et al. (2010). This technique allows the detection of gelatinase activity based on the digestion of gelatin embedded in polyacrylamide gels^31,32^.

Briefly, proteins were separated by electrophoresis under denaturing but non-reducing conditions using 10% SDS-polyacrylamide gels copolymerized with 0.1% gelatin (type A from porcine skin, Sigma-Aldrich). Equal volumes of protein samples were mixed with non-reducing sample buffer and loaded onto the gel without prior boiling. Electrophoresis was carried out at a constant voltage until the dye front reached the bottom of the gel.

Following electrophoresis, the gels were washed twice in 2.5% Triton X-100 solution for 30 min at room temperature to remove SDS and allow renaturation of the enzymes. The gels were then incubated in zymography incubation buffer (50 mM Tris-HCl, pH 7.4, 5 mM CaCl₂, 0.02% NaN₃) overnight at 37 °C to enable enzymatic digestion of the gelatin substrate.

After incubation, the gels were stained with 0.5% Coomassie Brilliant Blue R-250 in a solution containing 50% methanol and 10% acetic acid for 30–60 min, and then destained in 30% methanol and 10% acetic acid until clear bands representing gelatinolytic activity appeared against a dark blue background. Areas of enzymatic activity (MMP-2 and MMP-9) were visualized as clear lytic bands. Membranes were imaged and cropped for presentation after detection. No lanes were removed or rearranged. Original full-length blot images could not be retrieved due to archival limitations. All available images are presented in the figures, and replicates are described in the text.

### RNA extraction and cDNA synthesis

#### RNA extraction

Total RNA was isolated from whole blood samples using the GF-1 Total RNA Extraction Kit (Vivantis, Cat. No. GF-TR-100), following the manufacturer’s protocol. Blood cells were lysed using a proprietary lysis buffer that inactivates endogenous RNases. Samples were then passed through a homogenization column to remove cellular debris and genomic DNA, followed by on-column DNase I treatment to eliminate residual DNA. Selective RNA binding was achieved with ethanol-containing buffer, and RNA was eluted in RNase-free water.

#### RNA quantification and quality assessment

The concentration and purity of the extracted RNA were evaluated using a NanoDrop ND-1000 spectrophotometer. The A260/A280 absorbance ratio was used to assess RNA purity, with acceptable values between 1.8 and 2.0.

#### cDNA synthesis

Complementary DNA (cDNA) was synthesized using the Viva 2-Step RT-PCR Kit (Vivantis, Product No. RTPL12). First-strand synthesis was performed using M-MuLV Reverse Transcriptase (RNase H⁻) and either oligo(dT) primers or random hexamers, according to the manufacturer’s instructions. The absence of RNase H activity enhances the yield of full-length cDNA by preventing degradation of the RNA-DNA hybrid during the reverse transcription process.

### Quantitative real time PCR (qPCR)

PCR amplification of target genes was conducted using standard protocols. For gene expression analysis, quantitative real-time PCR (qPCR) was performed using the Maxima SYBR Green/ROX qPCR Master Mix (2X) (Thermo Scientific, Cat. No. K0221). Amplification and fluorescence detection were carried out on a real-time PCR system equipped with SYBR Green detection channels. This method allows for quantification of gene expression levels based on the fluorescence signal intensity during the exponential phase of PCR amplification (Kim et al. 2006). The reaction mixture was performed in a total volume of 20 mL containing 4 mL of cDNA (100 ng/mL), 300 nmol/L of each primer set for each gene, and 10 mL of SYBR Green Master Mix and completed to 20 mL with nuclease-free water. Each gene expression was normalized with the housekeeping gene GAPDH. The primer sequences for different genes: Glycdraldehyde-3-phosphate dehydrogenase (GAPDH), Heat shock proteins (HSP), DNA methyltransferase 3 A (DNMT3A), and interleukin 1-beta (IL1β), were synthesized by Biosearch Technologies, USA and listed in Table 2. The thermal cycler program was as follows: 95 ◦C for 5 min and 40 cycles of 94 ◦C for 15 s, annealing for 60 s according to melting temperature suitable for each primer set, and extension at 72 °C for 10 s. Quantitative Real-Time PCR (qRT-PCR) analysis was performed on RNA extracted from a total of 12 blood samples (6 infected and 6 non-infected sheep). Each reaction was run in triplicate (technical replicates). The 2-ΔΔCT formula, the method of relative quantification of mRNA was used to determine the fold difference in gene expression^33^.

**Table 2:** Primers sequences used in qRT-PCR.

Gene	Primer	Sequence (5`→3`)	Reference
HSP	Forward Reverse	CTTGGTCTTGGTATTGACGA AGAACATATTGGAGGGAACG	Developed by Primer-BLAST by using Primer-3 at NCBI^34^
DNMT-3 A	Forward Reverse	CCTGGCCTTATGGGCTGAGA TCATCGTCAGCTGCTTTGGT	
IL-1β	Forward Reverse	GTGCAGTCAGTAAAATGCAA GATTCTTGTCCCTGATACCC	
GAPDH	Forward Reverse	GTTTGTGATGGGCGTGAACC GGCGTGGACAGTGGTCATAA	

### Statistical analysis

The results obtained were statistically analyzed by using Model GLM of SAS software version (9.1) [NO_PRINTED_FORM]^35–38^. The Duncan Multiple Range test was used to test the level of significance among the means^39^. The difference was considered statistically significant at *P* < 0.05. Histograms and graphical presentations of the data were generated using GraphPad Prism version 8.

#### Summary of software and tools

A comprehensive list of the software and online tools used throughout the study is provided in Table 3.

**Table 3:** Software and online tools used in this study.

Software/tool	Purpose in study	Provider/references	URL
The concentration and purity	Primer design for qRT-PCR	NCBI^34^	https://www.ncbi.nlm.nih.gov/tools/primer-blast/
The concentration and purity	Sequence identity analysis with GenBank references	NCBI^40^	https://blast.ncbi.nlm.nih.gov/
The concentration and purity	Phylogenetic tree construction (NJ, ML, MP) with bootstrap analysis	Tamura et al.^41^	
The concentration and purity	Sequence alignment	DNASTAR, Madison, WI, USA^42^	https://www.dnastar.com/
The concentration and purity	Statistical analysis	SAS Institute Inc., Cary, NC, USA^43^	https://www.sas.com/
The concentration and purity	Graphing and histograms	GraphPad Software, San Diego, CA, USA^44^	https://www.graphpad.com/

## Results

### Anaplasmosis incidence with regards to microscopical detection by stained smears examination

**Fig. 2:**
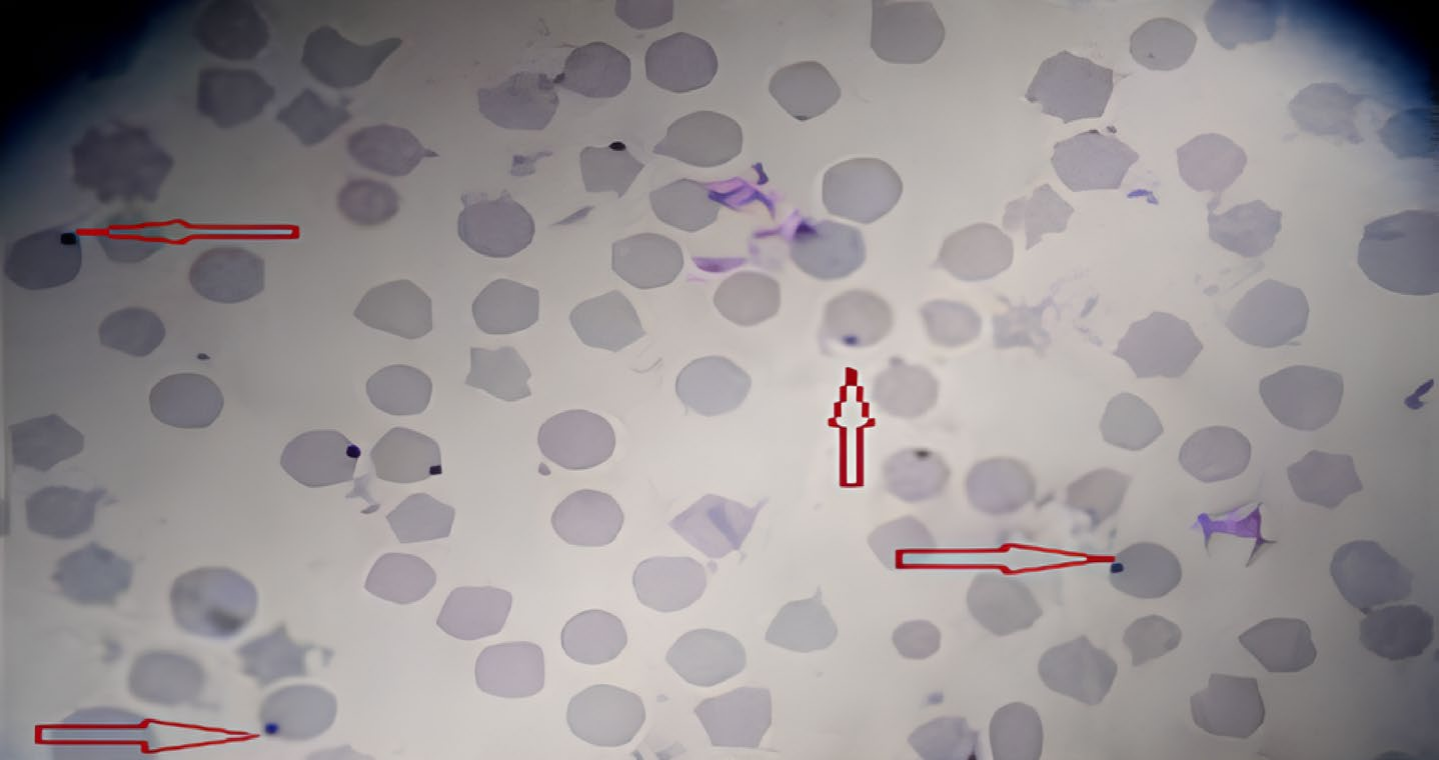
Microscopic examination of Giemsa-stained blood smear from a sheep infected with *Anaplasma marginale*. The intraerythrocytic inclusions (indicated by arrowheads) appear as distinct purple-stained bodies within the erythrocytes. (Giemsa stain, oil immersion, 100× magnification).

Microscopic examination of blood samples stained with Giemsa’s method showed that, out of the total 139 collected specimens, 106 were confirmed to be *Anaplasma marginale* with an overall prevalence of 76%. as demonstrated in Fig. 2.

### Anaplasmosis incidence with regards to PCR detection

**Fig. 3:**
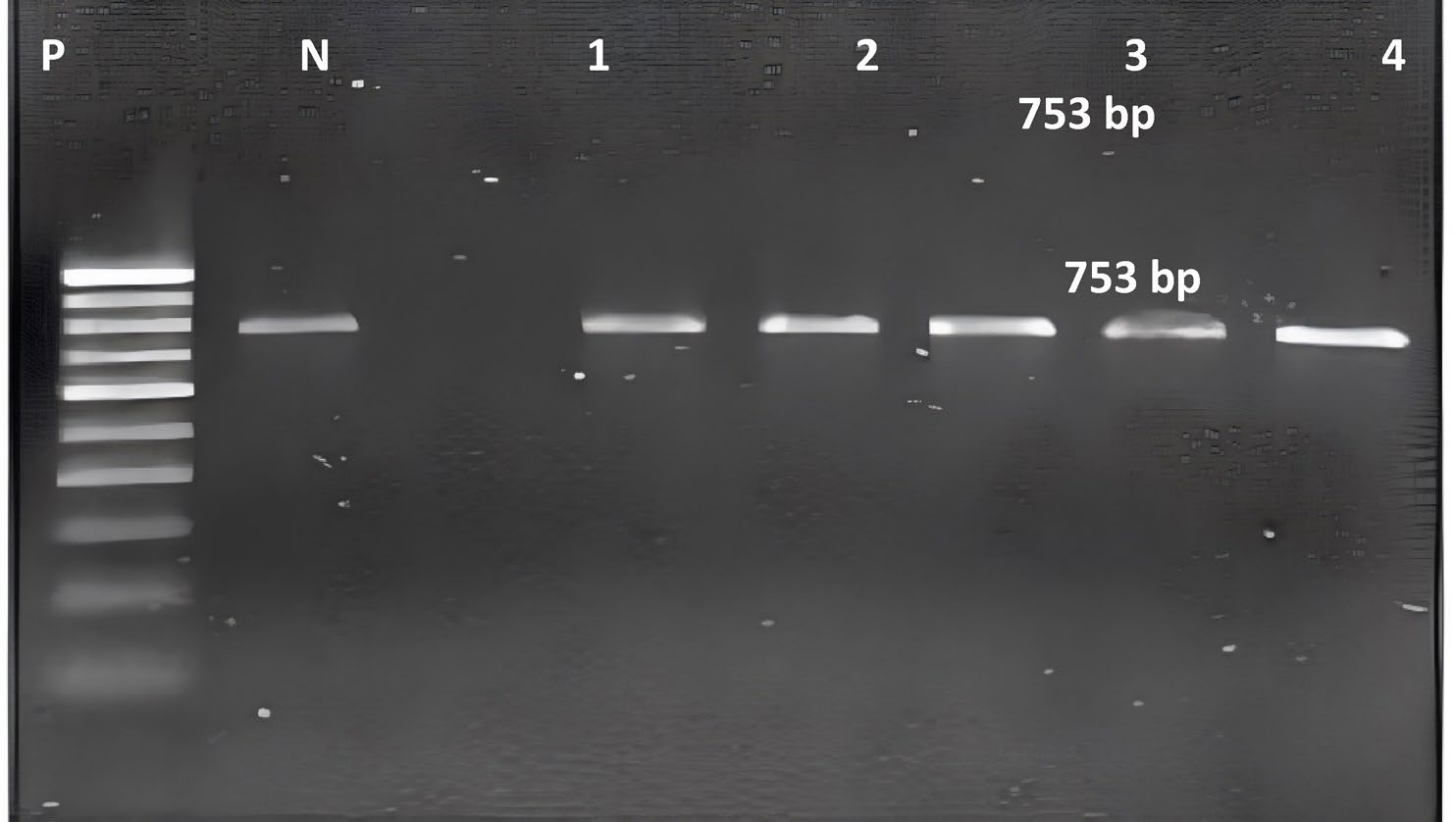
Molecular identification of *A. marginale by PCR on 1% agarose gel stained with ethidium bromide.* Lane M: DNA molecular size marker{Gene- ruler 100 bp ladder (Fermentas, Thermo) (100–1000 bp)}. N: negative control, P: positive control, Lane (1–5):753 bp amplicon of confirmatory msp4 gene for the 2 *A. marginale* stains in the examined samples. Note: The gel image was cropped to focus on the target amplicon bands. The original uncropped gel image is not available due to data archiving limitations at the time of image capture. No lanes were removed or rearranged, and the gel was processed intact.

**Fig. 4:**
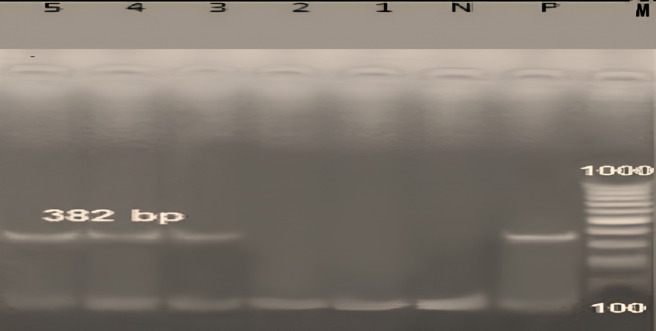
Molecular identification of *Anaplasma marginale by* PCR-product of *msp5 gene in 2% agarose gel stained with* Ethidium bromide. Lane M represents a DNA molecular size marker (GeneRuler 100 bp Ladder, Fermentas, Thermo, 100–1000 bp). N: negative control, P: positive control, 1–5: 382 bp amplicon of msp-5 gene in the examined samples. Note: The gel image was cropped to focus on the target amplicon bands. The original uncropped gel image is not available due to data archiving limitations at the time of image capture. No lanes were removed or rearranged, and the gel was processed intact.

The molecular identification of Anaplasma spp. by PCR revealed only one distinct species (*Anaplasma marginale) that* was detected in 79.85% of the sampled animals (Fig. 3). PCR analysis data revealed the obtaining of a specific band at the expected molecular size 382 bp of the target msp5 gene sequence, and an amplicon of 753 bp was noticed for msp4 gene (Fig. 4).

### Molecular characterization of Anaplasma marginale

**Fig. 5:**
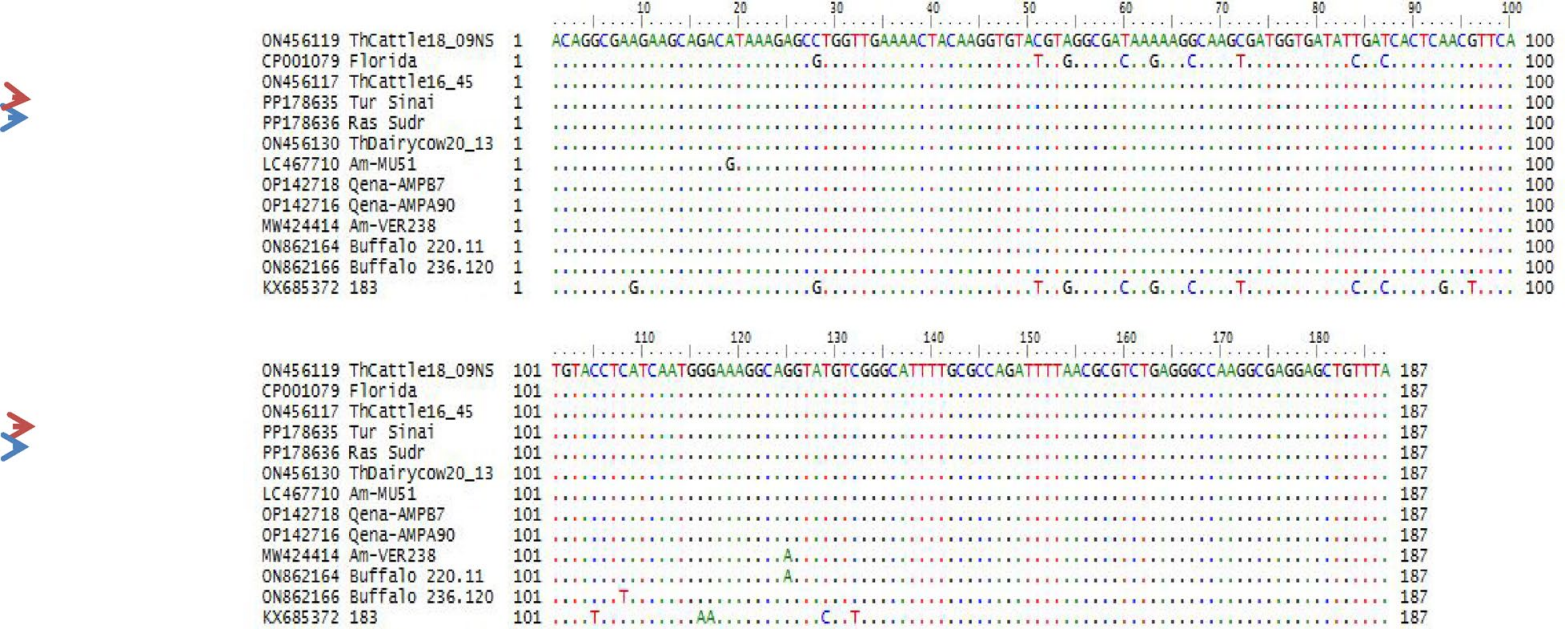
Alignment of the nucleotide sequences obtained from the present *Anaplasma marginale* isolates compared to reference sequences retrieved from the GenBank database. Sequence alignment was performed using the MegAlign module of Lasergene DNASTAR version 12.1. The two isolates from the present study are highlighted with red and blue arrows.

**Fig. 6:**
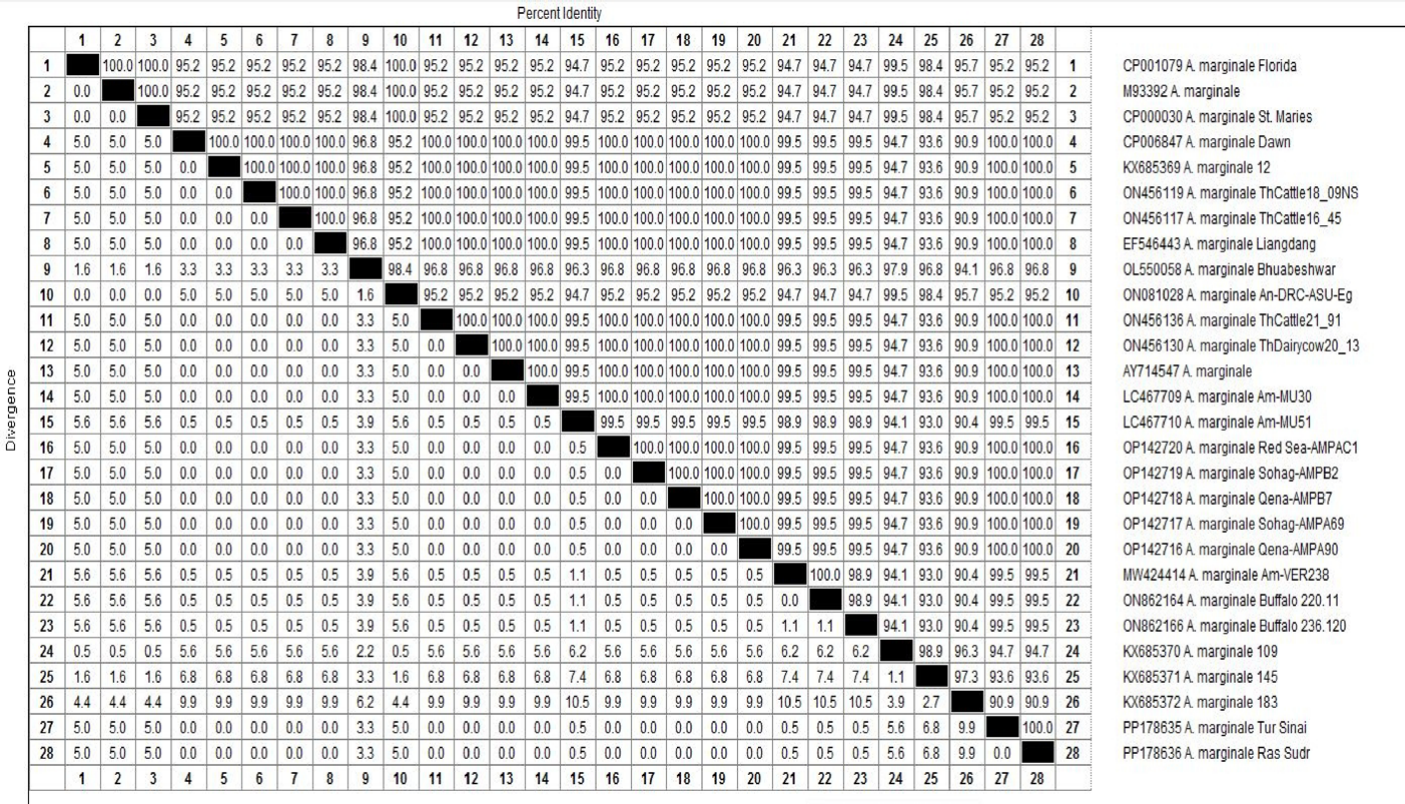
Pairwise sequence distance analysis of the *Anaplasma marginale* isolates created using the MegAlign module of Lasergene DNASTAR version 12.1. Sequence identities of 100% homology were recorded between two Egyptian A. marginale sequenced strains and Anaplasma marginale strains uploaded from gene bank public data.

**Fig. 7:**
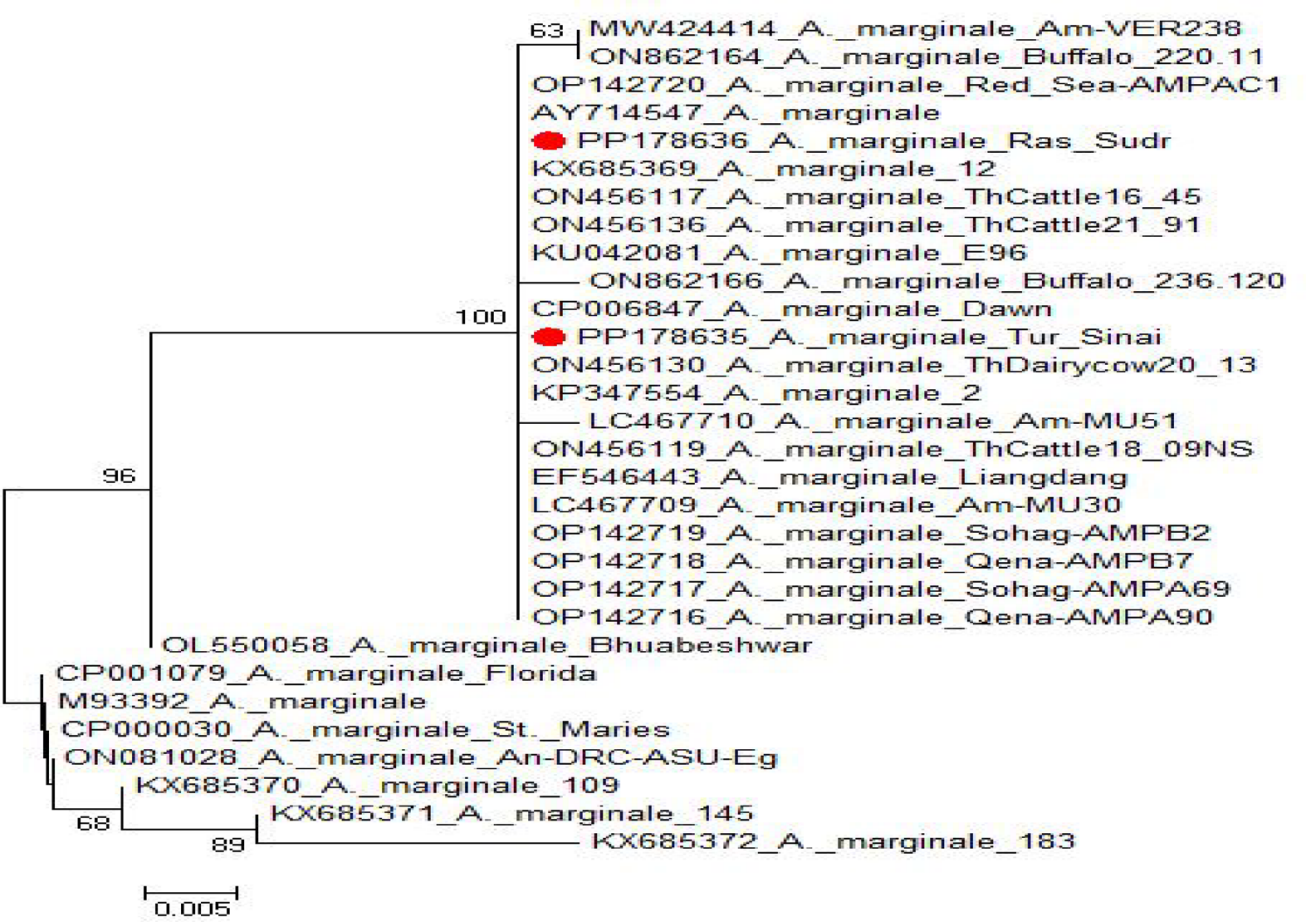
Phylogenetic tree illustrating the relationship between the sequenced *Anaplasma marginale* strains from this study and randomly selected reference strains from the GenBank database. The tree was constructed using the neighbor-joining and maximum parsimony methods implemented in MEGA6, with the Kimura 2-parameter substitution model and 1000 bootstrap replications. The two isolates from the present study are highlighted with red circles and show clear clustering with other *A. marginale* reference strains.

The sequence analysis of the MSP5 gene–PCR products identified two typical A. *marginale* that submitted to GenBank compared to the other *Anaplasma* preserved strains. The analysis of the two genetic loci showed no genetic diversity between them (identity percent between them is 100%), and their accession numbers and data were listed in Table 4 and (Fig. 5). They were identified under accession numbers: PP178635 and PP178636. They are clustered together with other different Anaplasma marginale previously submitted to GenBank data (Fig. 6). They are identical, showing complete nucleotide homology with each other, and the sequence identity between them is 100% with no different SNPs or divergent and 0.00 genetic distance. Their identities with other preserved strains range from 95.2 (Florida strain) to 100% (Dawn strain), and genetic distance from 0.00 to 9.9 as shown in (Fig. 7).

No nucleotide variation was observed between A. marginale sequences generated in this study with those reported previously from Egypt (OP142716 - OP142720)^45^. Kumar et al. (2019b), who used msp5 in the phylogenetic characterization of A. marginale isolates from Western India, have also reported a similar trend in the phylogenetic relationship of A. marginale wherein their sequences of msp5 gene were found identical to a fixed number of previously described sequences and divergent from the rest.

The sequences, generated in the present study, showed complete homology with those reported earlier from different governorates of Egypt (OP142716–OP142720). On the contrary, they were markedly different from those reported from Florida (CP001079).

In this study, both *msp4* and *msp5* genes were targeted for the molecular detection of *Anaplasma marginale*. However, PCR amplification of *msp4* did not yield sufficient product for downstream sequencing, likely due to low template abundance or suboptimal amplification. In contrast, the *msp5* gene consistently produced a clear 382 bp band, which was sequenced successfully. Due to the limited number of high-quality sequences obtained (*n* = 2), a phylogenetic tree could not be constructed from local sequences alone. Instead, the two sequences were compared with global *A. marginale* reference strains from GenBank. Phylogenetic analysis revealed that the local isolates clustered closely with known *A. marginale* strains, supporting their identification and geographical relevance.

**Table 4:** Identity and GenBank accession numbers of *Anaplasma marginale* DNA isolates obtained from sheep in the Sinai region of egypt.

No.	Host	Location	GenBank Accession Number and Isolate ID
1	Sheep	Tur Sinai	PP178635 (*A. marginale* Tur Sinai)
2	Sheep	Ras Sudr	PP178636 (*A. marginale* Ras Sudr)

#### Biochemical findings

##### Oxidative stress biomarkers

**Fig. 8:**
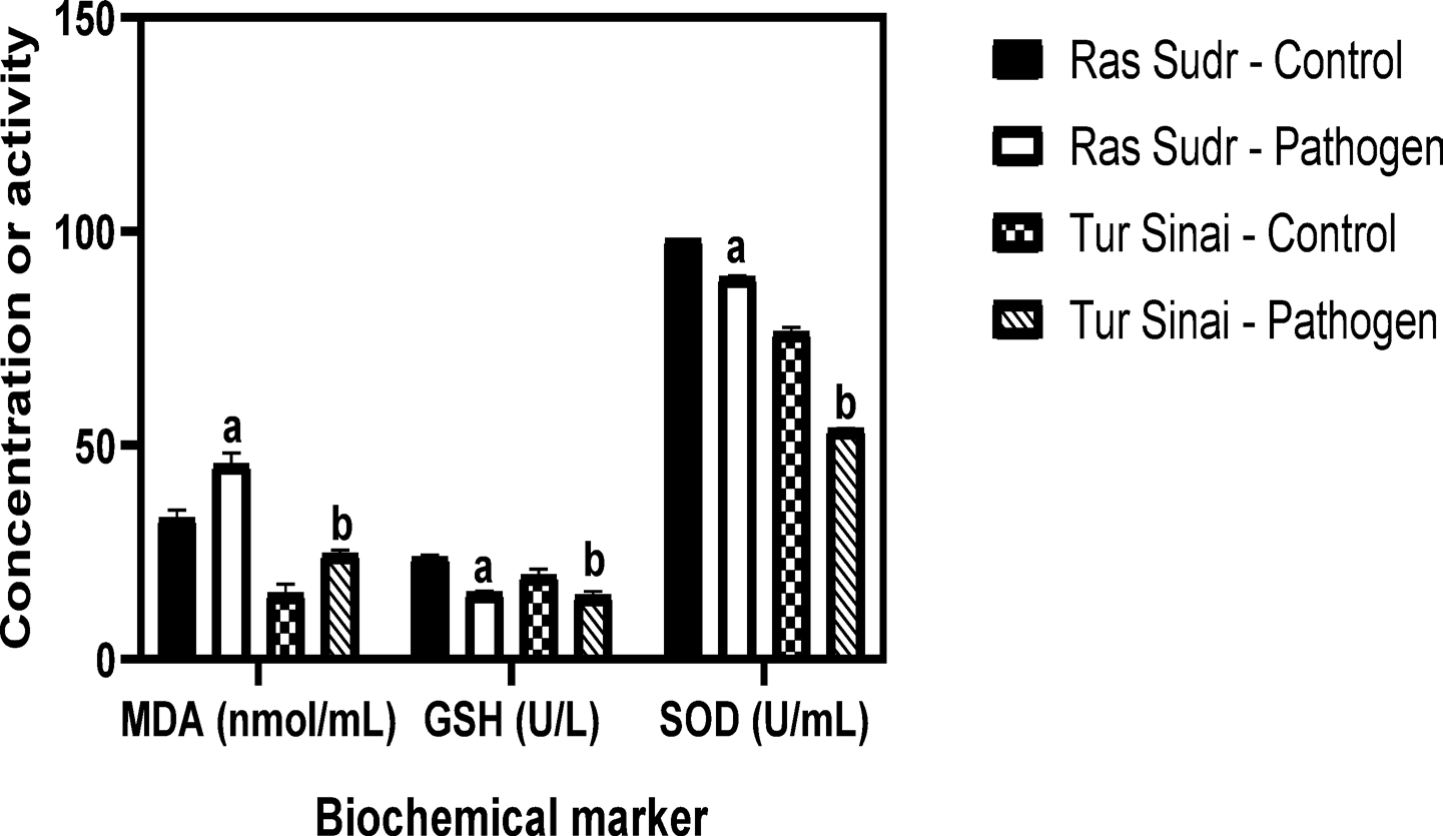
Comparison of the mean serum levels of malondialdehyde (MDA), reduced glutathione (GSH), and superoxide dismutase (SOD) activity between *Anaplasma* sp.-infected and non-infected sheep groups in Ras Sudr and El-Tur localities. Data are expressed as mean ± SD for *n* = 6 animals per group. Different letters indicate significant differences between groups (*P* < 0.05) based on Duncan’s Multiple Range Test using SAS **a**: significantly different from Ras Sudr control; **b**: significantly different from El-Tur control.

The concentration of MDA showed a significant (*p* < 0.05) increase in the infected group of Sudr and Al-Tur (45.54 nmol/mL & 24.62 nmol/mL) in comparison to their controls (32.90 nmol/mL & 15.26 nmol/mL). Also, the GSH level significantly (*p* < 0.05) dropped from 23.85 to 19.56 U/L in the non-infected control groups in Ras Sudr and Al-Tur to 15.54 & 14.87 U/L for the infected groups, respectively. However, the SOD activity was significantly (*p* < 0.05) decreased in infected groups of Sudr and Al-Tur to (89.26 U/mL & 53.82 U/mL) in comparison to their controls (98.1, 76.41) respectively as shown in (Fig. 8).

##### Matrix Metallo proteinases findings

**Fig. 9:**
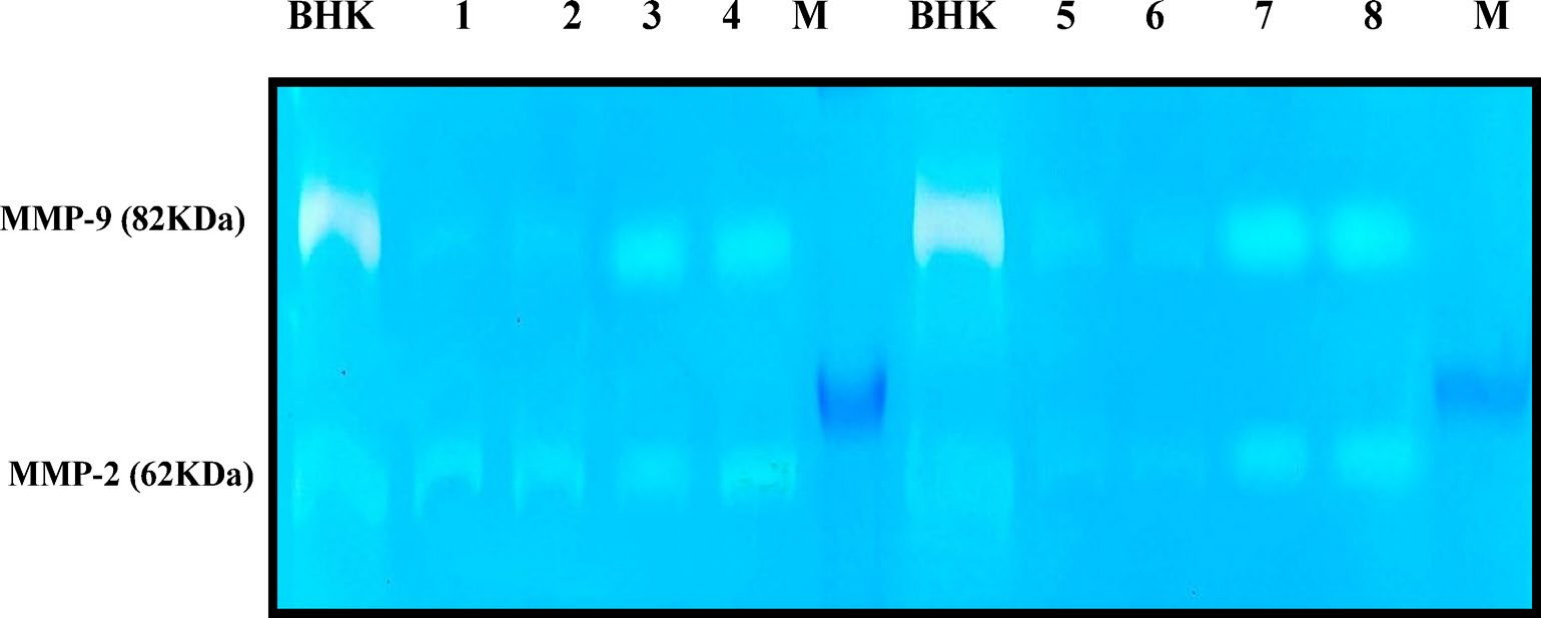
Gelatin zymography showing the enzymatic activity of gelatinases (MMP-9 and MMP-2) in *Anaplasma*-infected and non-infected samples from Ras Sudr and Al-Tur localities. Lanes 1–2: Non-infected control samples (Ras Sudr); Lanes 3–4: Infected samples (Ras Sudr); Lanes 5–6: Non-infected control samples (Al-Tur); Lanes 7–8: Infected samples (Al-Tur). BHK: Baby hamster kidney cells transfected with active MMP-9 (82 kDa) and MMP-2 (62 kDa) as positive control. M: Blue Eye Prestained Protein Marker (range: 10–245 kDa). Clear bands indicate proteolytic activity corresponding to the molecular weights of MMP-9 and MMP-2. Note: “Blots were cropped for clarity; full-length images were not retrievable due to archival limitations. No lanes were removed or altered.”

**Fig. 10:**
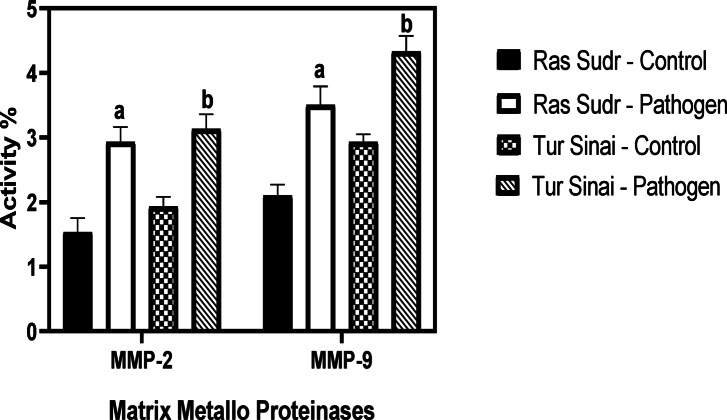
Comparison of the mean serum values of MMP-2 and MMP-9 activities between *Anaplasma* sp.-infected and non-infected sheep groups in Ras Sudr and El-Tur localities. Data are expressed as mean ± SD for *n* = 6 animals per group. Different letters indicate significant differences between groups (*P* < 0.05) based on Duncan’s Multiple Range Test using SAS (v9.1). **a**: significantly different from Ras Sudr control; **b**: significantly different from El-Tur control.

The activity of MMPs (2&9) was significantly (*P* < 0.05) increased in pathogenic groups of Sudr and Al-Tur in comparison to their control values as shown in Figs. 9 and 10.

##### Gene expression of HSP, DNAMT-3 A and IL-1ß

**Fig. 11:**
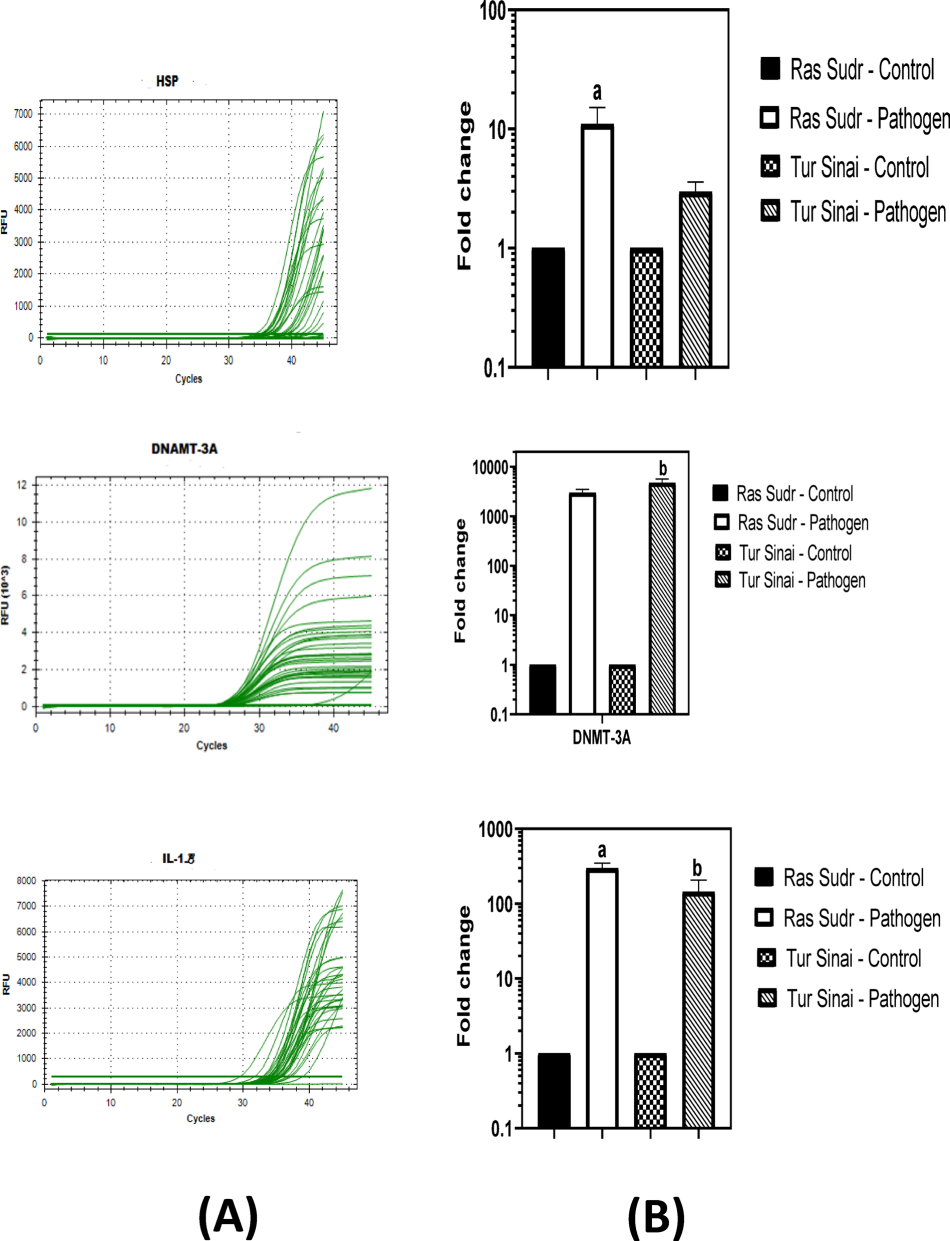
(**A**) Quantitative real-time PCR (qRT-PCR) amplification plots showing the relative expression levels of HSP, DNMT3A, and IL-1β genes in blood samples from Anaplasma-infected and non-infected sheep. (**B**) Comparative analysis of the mean gene expression values of HSP, DNMT3A, and IL-1β between infected (*n* = 6) and non-infected (*n* = 6) sheep groups from Ras Sudr and Al-Tur localities. Gene expression was normalized to the housekeeping GAPDH gene and analyzed using the 2^−ΔΔCt method. Data are expressed as mean ± SD for *n* = 6 animals per group. Different letters indicate significant differences between groups (*P* < 0.05) based on Duncan’s Multiple Range Test using SAS (v9.1). a: significantly different from Ras Sudr control; b: significantly different from El-Tur control.

The result of the present study showed that the expression level of the HSP gene was significantly (*P* < 0.05) increased in the infected animals of Sudr in comparison to the control group and its value is higher in the infected animals of Tur group compared to the control despite they are statistically non-significant. On the other hand, the expression of the DNAMT-3 A gene was significantly (*P* < 0.05) increased in the infected animals of Tur group in comparison to the control one and its value was higher in infected animals of Sudr in comparison to its control despite of they are statistically non-significant. It has been noted that the expression of the IL-1ß gene was significantly (*P* < 0.05) increased in both infected animals’ groups, due to inflammation, in comparison to the control, and the significance was more prominent in Sudr than El-Tur as shown in (Fig. 11).

## Discussion

The accurate and sensitive diagnosis of Anaplasma marginale in animals, along with rapid intervention, is critical to avoid economic loss. The present study investigated the prevalence and impact of *Anaplasma marginale* in sheep populations within Southern Sinai, Egypt. Microscopic examination revealed a high prevalence of 76% (106/139), aligning with previous reports of widespread *A. marginale* infection in livestock worldwide^46,47^. PCR analysis further confirmed infection in 79.85% of the sampled animals, emphasizing the need for sensitive diagnostic methods, as traditional microscopy can underestimate infection rates, particularly in carriers with low parasitemia^48^. This slight difference in prevalence between microscopy and PCR is expected and reinforces the importance of PCR for accurate diagnosis. Similar molecular detection in camels in Egypt reported distinct *A. marginale* strains using MSP4^49^, positioning our study as complementary to broader livestock surveillance.

To reach a higher diagnosis percentage, DNA sequencing of the MSP5 gene from two *A. marginale* isolates revealed high sequence identity (95.2–100%) with other strains, including the Florida strain from the U.S^50^. This finding aligns with previous studies demonstrating genetic diversity within *A. marginale* populations^51–53^. Phylogenetic analysis clustered these isolates with other *A. marginale* strains, further supporting their identification and suggesting a possible shared ancestry. The significance of MSP1–MSP5 in strain differentiation has been reinforced by recent genetic studies^17^.

Consistent with previous investigations into anaplasmosis in other ruminants^1,18,54–56^ this study found that *Anaplasma marginale* infection in sheep leads to significant changes in antioxidant enzyme activities. Specifically, we observed a decrease in SOD and GSH levels and an increase in MDA levels in infected animals. These findings reinforce the established role of oxidative stress in anaplasmosis pathogenesis^56–59^, indicating that *A. marginale* induces oxidative stress in sheep, mirroring its effects in other ruminant species. Prior research further supports this conclusion, with studies on *A. marginale*-infected calves^29,60^, *Babesia ovis*-infected sheep^61^ and *Anaplasma ovis*-infected goats^62^ all demonstrating compromised antioxidant status and increased oxidative stress markers.

Increased activity of matrix metalloproteinases (MMP-2 and MMP-9) in infected sheep suggests an inflammatory response, consistent with previous studies linking MMPs to various disease processes^63,64^. This suggested that MMPs may play a role in the pathogenesis of anaplasmosis, potentially contributing to tissue damage or remodeling.

The present study investigated the transcriptional responses of key genes, namely DNMT3a, IL-1β, and HSPs, in sheep infected with *A. marginale* and provide novel insights into the molecular mechanisms underlying the host-pathogen interaction and the immune response elicited during *A. marginale* infection in sheep. The significant upregulation of *DNMT3a* in infected sheep suggests an active role for DNA methylation in the host response. *DNMT3a* is a *de novo* DNA methyltransferase, meaning it establishes new methylation patterns, unlike *DNMT1* which primarily maintains existing patterns. Upregulation of *DNMT3a* could lead to significant and lasting changes in gene expression, potentially affecting immune responses, cell cycle regulation, and overall host defense mechanisms^65–67^*De novo* methylation by DNMT3a is critical in development and cellular differentiation, but its role in immune responses is increasingly recognized. The observed increase suggests that *A. marginale* infection might be inducing a reprogramming of the host’s gene expression through *DNMT3a* activity. Further investigation is needed to identify the specific genes targeted by this increased *DNMT3a* activity and to understand the functional consequences of these methylation changes. It would be exciting to investigate if *DNMT3a* targets genes involved in the innate or adaptive immune response to *A. marginale*.

The observed upregulation of IL-1β (as shown in Fig. 11B) in infected sheep is consistent with its established role as a key pro-inflammatory cytokine. IL-1β plays a crucial role in initiating and amplifying the inflammatory response, recruiting immune cells to the site of infection, and promoting pathogen clearance. However, excessive IL-1β production can also contribute to tissue damage and systemic inflammation, as observed in various infectious diseases. These findings are consistent with previous reports on the role of IL-1β in sheep during infection^68–70^.

The significant up-regulation of HSPs in infected sheep indicates cellular stress responses. HSPs are molecular chaperones that play critical roles in protein folding, refolding, and degradation and are induced in response to various stressors, including infections. The increased expression of HSPs in infected sheep likely reflects the cellular stress associated with *A. marginale* infection, such as oxidative stress, inflammation, and cellular damage, which is consistent with previous studies linking HSPs to cellular stress during infection^71–74^.

Although both *msp4* and *msp5* genes were targeted for molecular detection of *Anaplasma marginale*, only the *msp5* gene yielded high-quality PCR products suitable for sequencing. The *msp4* gene did not amplify sufficiently, despite multiple attempts, and therefore could not be included in the phylogenetic analysis due to the inadequate quantity and quality of the PCR product.

Furthermore, while sequencing the *msp5* amplicons was successful, only two sequences from local isolates met the required quality standards and were submitted to GenBank (Accession Nos. PP178635 and PP178636). Given the limited number of sequences, construction of a statistically robust phylogenetic tree using laboratory-obtained isolates alone was not feasible. Instead, these sequences were compared with global reference strains from the GenBank database. Phylogenetic analysis using neighbor-joining and maximum parsimony methods demonstrated that the local isolates clustered closely with other known *A. marginale* strains, confirming their genetic relatedness and supporting their identification.

These molecular findings, when integrated with the laboratory assessments of oxidative stress biomarkers, gene expression data, and morphological confirmation, offer a comprehensive understanding of the infection dynamics. The consistency between the molecular and physiological data underscores the relevance of the study and highlights the regional epidemiology of *A. marginale* infections in small ruminants.

While the diagnostic and molecular techniques used in this study are well established, their integrated application to naturally infected sheep in Egypt, supported by transcriptional and oxidative stress profiling, provides a novel regional insight. These findings contribute to a more localized understanding of the disease dynamics and may inform control strategies tailored to endemic regions.

In conclusion, Molecular characterization provides valuable insights into the genetic diversity of *A. marginale* circulating in the region. The observed changes in gene expression provide valuable insights into the molecular mechanisms underlying anaplasmosis in sheep, including the role of epigenetic modifications, inflammation, and cellular stress responses. The differential expression of genes such as IL-1β and HSPs could potentially serve as biomarkers for the diagnosis and prognosis of anaplasmosis, though further research is needed in this area. The observed oxidative stress and immune responses highlight the significant impact of *A. marginale* infection on sheep health.

This research emphasizes the importance of implementing effective disease control strategies to mitigate the impact of anaplasmosis on sheep production in Egypt.

## Data Availability

The datasets generated and/or analysed during the current study are available in the NCBI GenBank repository, under accession numbers PP178635 (https://www.ncbi.nlm.nih.gov/nuccore/PP178635) and PP178636 (https://www.ncbi.nlm.nih.gov/nuccore/PP178636).

## Abbreviations

± SE: Standard Error
µL: Microliter
ARRIVE guidelines: Animal Research: Reporting of in Vivo Experiments
ARM: age-related Maculopathy
BA: Bovine Anaplasmosis
BHK: Baby hamster kidney
BLAST: Basic Local Alignment Search Tool
bp: Base pair
CAT: Catalase
cDNA: Complementary DNA
CNV: Choroidal NeoVascularization
DNA: Deoxyribonucleic acid
DTNB: 5,5́́’ Dinitro-benzoic acid
EDTA: Ethylene diamine tetra acetic acid
GSH: Reduced glutathione
IACUC: Institutional Animal Care and Use Committee
IL-1β: Interlukine 1 beta
kDa: Kilodalton
MAb: Monoclonal antibody
MDA: Malondialdehyde
MMP-2: Gelatinase A
MMP-9: Gelatinase B
MMPs: Matrix metallo-proteinases
msp5: Major surface protein 5
nm: nanometer
PCR: polymerase chain reaction
RBC: Red Blood Corpuscles
RNA: Ribonucleic acid
ROS: Reactive Oxygen Species
SNPs: Single nucleotide polymorphisms
SOD: Super oxide dismutase
TBA: Thiobarbituric acid
TIMPs: Tissue Inhibitors of Metallo-Proteinases
TNF-α: Tumor necrosis factor alpha
UV: Ultraviolet
